# Origin of multiple periodicities in the Fourier power spectra of the Plasmodium falciparum genome

**DOI:** 10.1186/1471-2164-12-S4-S4

**Published:** 2011-12-22

**Authors:** Miriam CS Nunes, Elizabeth F Wanner, Gerald Weber

**Affiliations:** 1 Department of Biological Sciences, Federal University of Ouro Preto, 35400-000 Ouro Preto, MG, Brazil; 2 Department of Computer Engineering, Federal Center for Technological Education of Minas Gerais, 30421-169 Belo Horizonte, MG, Brazil; 3 Department of Physics, Federal University of Minas Gerais, 31270-901 Belo Horizonte, MG, Brazil

## Abstract

**Background:**

Fourier transforms and their associated power spectra are used for detecting periodicities and protein-coding genes and is generally regarded as a well established technique. Many of the periodicities which have been found with this method are quite well understood such as the periodicity of 3 nt which is associated to codon usage. But what is the origin of the peculiar frequency multiples *k*/21 which were reported for a tiny section of chromosome 2 in *P. falciparum*? Are these present in other chromosomes and perhaps in related organisms? And how should we interpret fractional periodicities in genomes?

**Results:**

We applied the binary indicator power spectrum to all chromosomes of *P. falciparum*, and found that the frequency overtones *k*/21 are present only in non-coding sections. We did not find such frequency overtones in any other related genomes. Furthermore, the frequency overtones were identified as artifacts of the way the genome is encoded into a numerical sequence, that is, they are frequency aliases. By choosing a different way to encode the sequence the overtones do not appear. In view of these results, we revisited early applications of this technique to proteins where frequency overtones were reported.

**Conclusions:**

Some authors hinted recently at the possibility of mapping artifacts and frequency aliases in power spectra. However, in the case of *P. falciparum* the frequency aliases are particularly strong and can mask the 1/3 frequency which is used for gene detecting. This shows that albeit being a well known technique, with a long history of application in proteins, few researchers seem to be aware of the problems represented by frequency aliases.

## Background

The detection and analysis of repetitions in genomes is one of the most recurrent problems in computational biology. It is of technological importance since it imposes significant limitations to next-generation sequencing technologies [1] and is related to numerous properties of the genome [2-5]. As a consequence it has sparked many approaches to detect and visualise genomic repeats [6]. Discrete Fourier transform (DFT) is one of this approaches, which is employed for the detection of genome wide non-exact periodic structures such as tandem repeats. In essence, the symbolic genome sequence is translated into numeric series which are then handled as a biological signal. The method was originally proposed for detecting periodicities in proteins [7-11] but has since gained popularity for genomes [12-18]. The genome of *Plasmodium falciparum*, the malaria parasite, is quite remarkable: it has an unusually high AT-content (80% or more) [19] and lacks identified transposable elements [20]. Sharma *et al*. [21] reported a complete frequency comb of all overtones of the basic frequency 1/21 nt^–1^ for chromosome 2 of *P. falciparum*, that is, all frequencies of type *k*/21 nt^–1^ with *k* = 1, 2, …, 10. Could this be yet another peculiarity of the *P. falciparum* genome? Such a frequency comb envisages several questions such as its biological origin and function, that were not addressed by Sharma *et al*. [21] and have remained unanswered, to the best of our knowledge. Additionally, it also gives rise to an important conceptual question: how should we understand fractional periodicities such as 21/2 or 21/4 given that there are no fractional nucleotides in our sequence? Or could it be that these multiples are so called frequency aliases [22,23]? In this work we analyse these questions more closely. First, we apply the Fourier transform to all chromosomes of *P. falciparum* and confirm that the 1/21 nt^–1^ overtone frequency comb is found in all but one chromosomes and present its location in the genome. But perhaps most importantly, we have established that this frequency comb is indeed composed of frequency aliases, and as such is an artifact of the way the genome is converted from a symbolic into a numeric sequence. While this resolves our current conceptual problem, it poses an important warning for the blind use of this technique, especially if used to detect genes. Should one of the frequency multiples coincide with the 1/3 frequency this could lead to important problems with the detection of coding regions [18,24-27]. We show that this is indeed the case for the 7/21 frequency of *P. falciparum*, which could cause false positive candidates for coding regions.

## Results and Discussion

**Figure 1:**
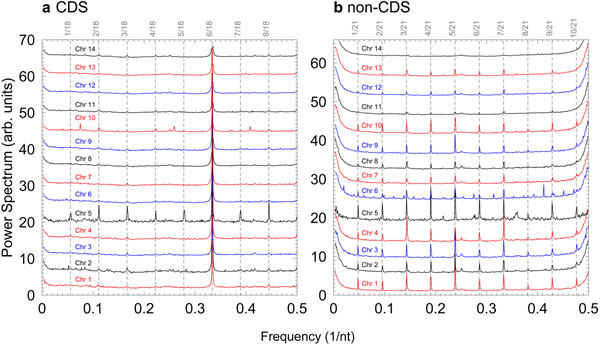
**Binary indicator power spectra for all chromosomes of *P. falciparum.*** The spectra were calculated separately for a) coding and b) non-coding region. The overlapping of the 1/3 peaks results from enlarging the vertical axis scale to allow visualising details of non-1/3 peaks. Vertical dashed lines show the positions corresponding to a) *k*/18 and b) *k*/21 frequency multiples. For clarity the curves were shifted vertically by arbitrary amounts.

In Fig. 1 we present the binary indicator power spectra for all chromosomes of *P. falciparum* separated by coding (CDS) and non-coding (non-CDS) regions. The *k*/21 frequency comb can be seen only for the non-coding sections of chromosomes 1–13 in Fig. 1b and is notably absent in chromosome 14. No chromosomes show this particular frequency comb in the coding section, Fig. 1a. However, chromosome 5 displays a slightly different frequency comb of *k*/18. Strong 1/3 nt^–1^ peaks in Fig. 1a and the lack thereof in Fig. 1b confirms that this genome appears to be well annotated [18,24-27].

**Figure 2:**
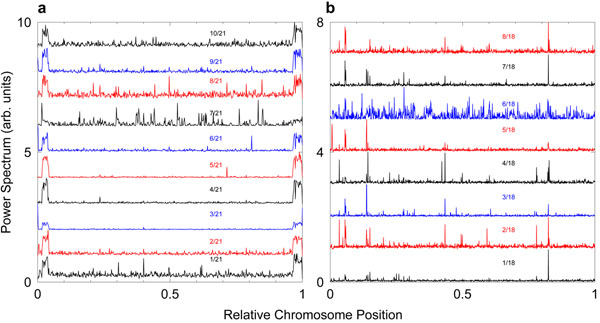
**Position-dependent power spectra of *P. falciparum.*** Part a) is for chromosome 1 and its *k*/21 frequency multiples and b) is for chromosome 5 and *k*/18 frequency multiples. A sliding window of ***w*** = 1000 nt was used. For clarity the curves were shifted vertically by arbitrary amounts.

Further refining the origin of the *k*/21 frequency comb shows that it comes mostly from regions at the extremes of the chromosomes, as shown in Fig. 2a, where we indicate the position dependent power spectra intensities for each of the *k*/21 overtones in chromosome 1. In contrast, the *k*/18 frequency comb originates from various regions of the genome as one would expect for CDS regions.

To identify the actual sequences involved in the *k*/21 frequency overtones, we extracted the first and last 4000 nt of each chromosome and analysed the repetitions with Tandem Repeats Finder [28]. The consensus sequences are shown in table 1, which indicates single occurrences of dimers CC or GG. For instance, chromosome 1 has one GG dimer, which is the only occurrence of guanine in this sequence. Therefore, this dimer will repeat with frequency 1/21 nt^–1^ and explains the fundamental frequency 1/21 nt^–1^ which we observe in Fig. 1b.

**Table 1:** Consensus sequences for periodicity 21 nt.

Chromosome	Relative Position	Consensus sequence	Repetitions
1	0.0179–0.0383	CTTAACTAACATAGGTCTTAA	622.8
	0.965–0.997	AGTTAGTTAAGTTAAGACCTA	994.3
2	0.0146–0.0223	ACTAACTTAGGTCTTAACTTA	348.2
	0.981-0.986	TTAGTTAAGTTAAGACCTAA^†^	264.3
3	0.0126–0.0236	GGTCTTACTTCACTAACATA^†^	552.2
	0.0157–0.0234	ATAGGTCTTAACTTAACTAAC	417.6
	0.0121–0.0283	TAGGTCTTAATTAACTAACT^†^	817.5
	0.986–0.988	TAAGACCTAAGTTAGTGAAGT	78.6
4	0.0139–0.0221	AGGTCTTACTTCACTAACTT^†^	472.4
	0.986–0.991	TTAGTTAAGTTAAGACCTAAG	238.2
5	0.0087–0.0129	ACTAACATAGGTCTTACTTC^†^	269.8
6	0.977–0.979	TAAGACCTATGTTAGTAAAG^†^	152.7
	0.982-0.990	AAAGTTAAGACCTAAGTTAGT	581.6
7	0.991–0.999	TTAAGACCTAAGTTAGTGAAG	543.8
8	0.0077–0.0119	ACTAGGTCTTAACTTAACTA^†^	269.6
	0.988–0.991	AAGACCTAAGTTAGTAAGTT^†^	185.4
9	0.978–0.985	GACCTATGTTAGTAAAGTAA^†^	551.6
	0.987–0.993	TAAGACCTAAGTTAGTGAAG^†^	413.4
10	0.0073–0.0116	TAGGTCTTACTTTAACTAACT	344.4
	0.981–0.990	GACCTATATTAGTTAAGTTAA	737.5
11	0.0072–0.0093	TTACTAACATAGGTCTTAAC^†^	202.3
12	0.0049–0.0055	TAACTTAGGTCTTAACTTCAC	67.5
	0.991–0.995	TATGTTAGTTAAGTTAAGACC	354.0
13	0.0049–0.0069	ACTAACATAGGTCTTACTTC^†^	269.8
	0.990–0.991	AGTAAGACCTTAGTTAGTGA^†^	192.5
	0.991-0.993	GACCTATGTTAGTGAAGTTAA	345.2

Main tandem repetitions of periodicity 21 nt found in the *P. falciparum* genome, except for chromosome 14 from which no such periodicities were detected. ^†^Some consensus sequences provided by Tandem Repeats Finder [28] are of size 20 nt, yet they all correspond to the periodicity of 21 nt.

However, we still do not know where the multiples of this frequency originate. The answer to this lies in a closer inspection of the repeated consensus sequences in table 1. Let’s take sequence TAAGACCTATGTTAGTAAAG for chromosome 6 which has a double cytosine. The four binary indicator sequences which result from converting the symbolic sequence are shown in table 2. The binary sequence for cytosine in table 2 displays two consecutive ones followed by zeroes only. In other words, we have two digits one followed by 19 zeroes, repeated hundreds of times. This is essentially analogous to a Fourier integral of a train of Kronecker delta functions, that is, a sequence of pulses with period *T*, which can be shown to result in another train of Kronecker delta functions with period 1/*T* in frequency space [29]. Therefore, the overtones *k*/21 do not correspond to real periods 21/*k* but are just artifacts of the way the symbolic sequence was mapped into binary sequences. Therefore, the frequencies 2/21, 3/21 …, 10/21 are frequency aliases of the frequency 1/21 [22,30].

**Table 2:** Example of sequence binary mapping.

	T	A	A	G	A	C	C	T	A	T	G	T	T	A	G	T	A	A	A	G
*n*	0	1	2	3	4	5	6	7	8	9	10	11	12	13	14	15	16	17	18	19
*u*_*A*,*n*_	0	1	1	0	1	0	0	0	1	0	0	0	0	1	0	0	1	1	1	0
*u*_*C*,*n*_	0	0	0	0	0	1	1	0	0	0	0	0	0	0	0	0	0	0	0	0
*u*_*G*,*n*_	0	0	0	1	0	0	0	0	0	0	1	0	0	0	1	0	0	0	0	1
*u*_*T*,*n*_	1	0	0	0	0	0	0	1	0	1	0	1	1	0	0	1	0	0	0	0

**Figure 3:**
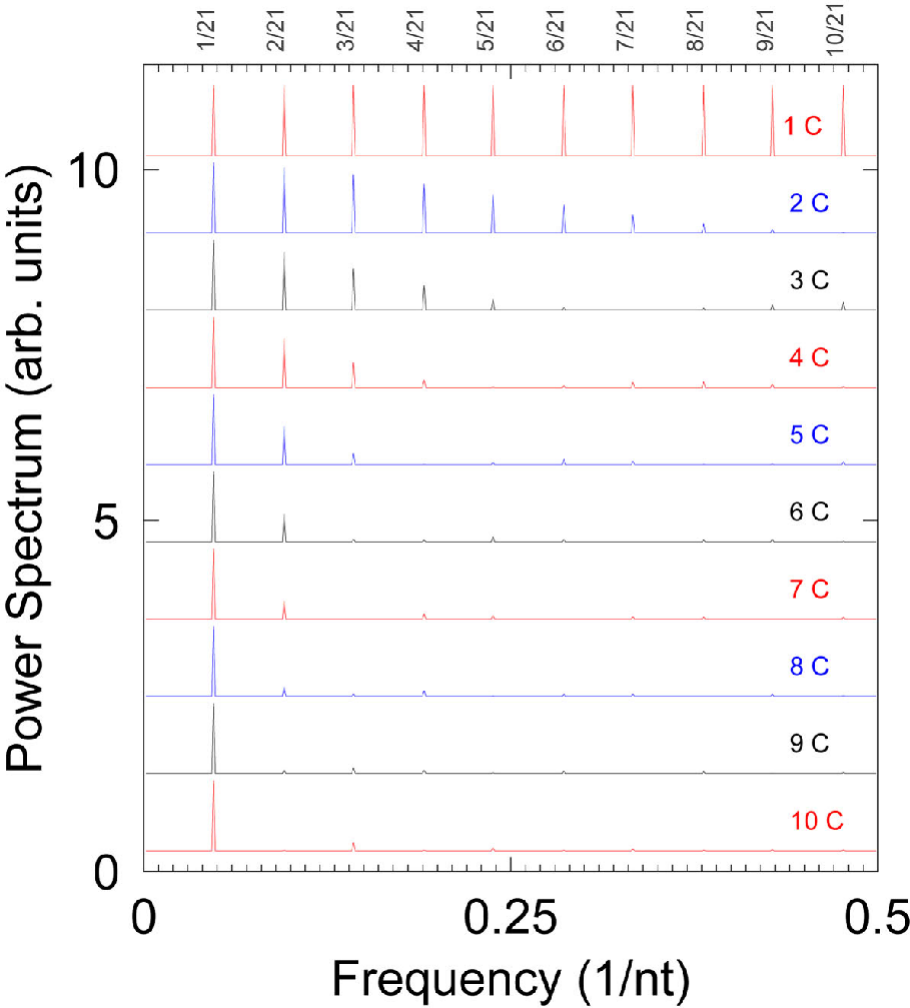
**Binary indicator power spectra for an artificially constructed sequence.** The sequence was constructed to contain 80 repeated units of size 21 nt. The spectra are for cytosine only (*α* = *C*) with varying numbers of consecutive cytosine nucleotides while keeping the periodicity constant at 21 nt.

In Fig. 3 we illustrated this effect by building an artificial sequence made of 80 repetitions of the basic unit of size 21 nt containing one or more consecutive cytosines. For ten cytosines we obtain exactly one peak at 1/21 nt^–1^ as expected. However, as we gradually reduce the number of consecutive cytosines, while maintaining the size of the repeating unit constant to 21 nt, we also obtain more of the *k*/21 overtones. Eventually, for one or two cytosines, all overtones *k*/21 are present, that is, we obtain all fractional periodicities although only one cytosine repeats every 21 positions.

**Figure 4:**
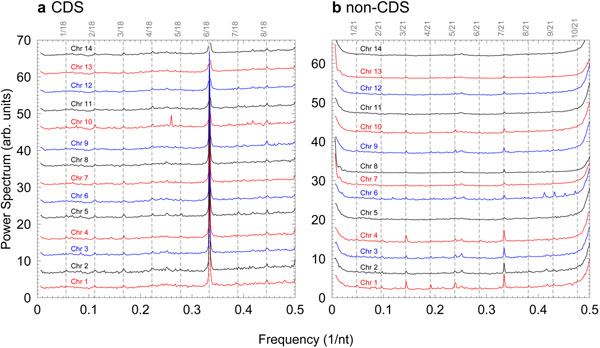
**Flexibility power spectra for each chromosome of *P. falciparum.*** Part a) is for coding regions and b) for non-coding regions. Also shown are the vertical dashed lines for the frequency multiples which are detected in Fig. 1. For clarity the curves were shifted vertically by arbitrary amounts.

One simple way to verify if these overtones are artifacts of the numerical mapping is to change the mapping. As an example, we computed the power spectra using DNA flexibility mapping, as detailed in Methods section. In this case the symbolic sequence is converted into a single numerical sequence and instead of using only zeroes and ones we have now ten different numerical values. This particular mapping was selected because converting di-nucleotides allows us to choose over a larger set of numerical values resulting into an overall smoother numerical profile along the sequence. Therefore the occurrence of delta-like structures in the numerical sequence should be less likely. The power spectra for both coding and non-coding sections of *P. falciparum* are shown in Fig. 4. As expected, the frequency overtones are now barely noticeable for a few chromosomes only. Most chromosomes show little or no overtones at all. We have established that the occurrence of overtones is the result of one or two isolated nucleotides surrounded by a large number of different nucleotides and very frequently repeated. But what is the minimal size of the repeating unit for which overtones may start to appear? We carried out numerical tests using a simple repeating unit CA*_n_* with *n* = 1, 2, …, 9, corresponding to a periodicity of *p* = *n* + 1 (data not shown), and overtones were present in all cases.

## Conclusions

**Figure 5:**
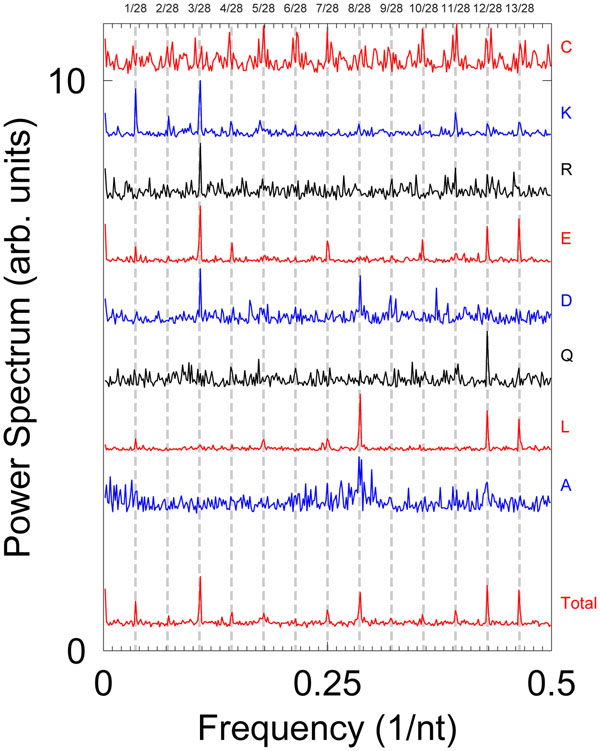
**Binary indicator power spectra of *Dictyostelium discoideum* myosin heavy chain gene.** In addition to the total spectrum, we show the individual spectra for amino acids with significant frequency harmonics. Vertical dashed lines are the positions of the *k*/28 frequency multiples. The curves were shifted vertically by arbitrary amounts for clarity.

It was suggested that the mapping of the symbolic sequence into one or more numeric sequences could give rise to artifacts when signal processing techniques are applied [31]. In this case, the frequency comb results as an artifact of dividing one symbolic sequence into four separate sequences, that is, it is an artifact of the multichannel analysis. Indeed, for proteins fractional periodicities were already observed in the pioneering work by McLachlan and Stewart [7,9]. The sequence mapping used some amino acid properties and is similar to the flexibility mapping outlined in the Methods section. In this case, the authors merely state that the periodicity does not need to be an integer value [7,9]. While non-integral periods for certain mappings may be plausible, multiple overtones are harder to explain in this way. Subsequent works, such as McLachlan *et al. *[32], explicitly use the multichannel mapping and clearly show frequency combs, see Fig. 3 of [32]. Since only total spectra were published [32], we recalculated the multichannel Fourier transform for *Dictyostelium discoideum* for each amino acid. Fig. 5 shows the total Fourier spectrum and the detailed spectra for some amino acids with important contributions towards the frequency comb. We confirmed that the frequency comb is the same type of frequency alias as seen for the genome of *P. falciparum.* For instance, glutamic acid (E) alone is sufficient to account for the *k*/28 multiples of the power spectra presented by McLachlan *et al. *[32].

While nucleotide binary indicator mapping separates the symbolic genomic sequence into four binary sequences, they are separated into 20 sequences for amino acids. Therefore, the likelihood that an amino acid appears repeatedly and in isolation, is much higher than for nucleotides. It is therefore not surprising that for almost every work we encountered, power spectra in amino acid sequences were reported with multiple frequencies when binary mapping was used [7-9,32-35]. What remains somewhat of an open question is the absence of any explanation for the frequency multiples and fractional periodicities in all these works.

The presence of strong frequency aliases in the power spectra of so many chromossomes is presently unique to *P. falciparum.* However, considering the accelerated pace of genomic sequencing, we cannot rule out further occurrences of such frequency aliases in other genomes. In this work we stress the need for a careful evaluation of frequency multiples if they appear in Fourier power spectra since most available bioinformatics applications do not distinguish these aliases from genuine frequencies.

## Methods

### Binary indicator sequence

The original nucleotide sequence of length *N* is converted into four numerical sequences of binary indicators,(1)

where the coefficient *u*_α,*n*_ indicates the presence or absence of a nucleotide of type *α* at position *n* with 1 or 0. This method is also sometimes called multichannel Fourier analysis where each *α* represents a channel [32].

For instance the sequence TAAGACCTATGTTAGTAAAG would be represented as shown in table 2. This numerical representation removes any content bias of the original genomic sequences which may be an advantage or not depending on its intended applications [18,36].

For a sequence of amino acids this mapping is trivially extended by mapping the 20 amino acids into the same amount of binary indicator sequences [32].

### Elastic constants

**Figure 6:**
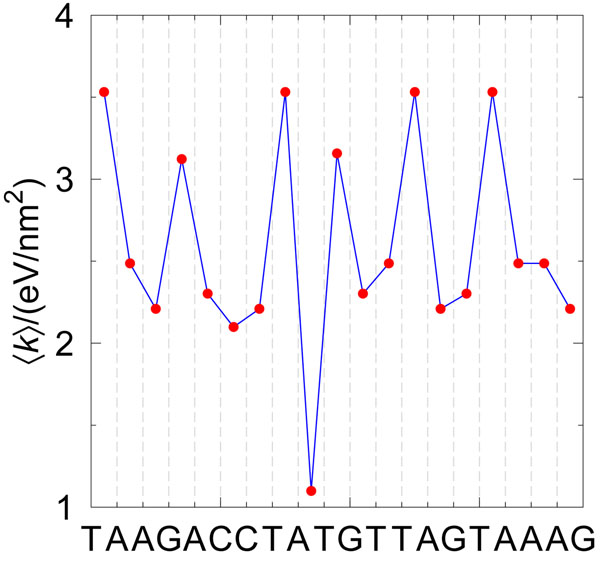
**Example of flexibility profile.** Each red bullet represents the flexibility of a given nearest-neighbour base-pairs of the sequence TAAGACCTATGTTAGTAAAG. For instance, the first is the flexibility of TpA, the second of ApA and so forth. Lines are intended as guide to the eye.

An alternative to the binary indicator sequence is to convert the genomic DNA sequence into a single numerical sequence using a conversion table. In this work, we use microscopic flexibilities for DNA calculated recently [37]. Instead of converting single nucleotides we convert pair-wise nearest-neighbour dimers following table 3. In this case the sequence TAAGACCTATGTTAGTAAAG would be represented by the flexibility profile shown in figure 6.

**Table 3:** Flexibilities of the ten unique nearest-neighbours dimers in DNA [37].

dimer step	*k* (eV/nm^2^)	dimer	*k* (eV/nm^2^)
ApA	2.40	ApC	2.56
ApG	2.25	ApT	1.83
CpA	3.44	CpC	2.06
CpG	2.74	GpA	2.80
GpC	3.36	TpA	2.42

### Power spectrum

For the binary indicator sequences we calculate the discrete Fourier transform (DFT) for each channel independently(2)

where *N* is the length of the sequence, and then combine the four resulting power spectra [24](3)

The DFT was implemented through the use of the efficient FFTW3 package [38].

For the flexibility profile we calculate only one Fourier transform and its corresponding power spectra(4)

where *M* is the length of the nearest-neighbour sequence and *M* = *N* – 1.

### Position dependent power spectrum

To identify the genomic origin of a given frequency *f* we apply the Fourier transform only to a section of size *w* starting at position *p* of the genome and divide this section along the genome monitoring only this specific frequency. In this way we are able to pinpoint the genomic sections which provide the strongest contribution to a specific frequency *f* = *l*/*w*. For the case of binary indicators this means taking Eq. (2) only over a window *w*(5)

and then monitoring a specific frequency *f* = *l*/*w*. In this work we used a window of size *w* = 1000 nt, unless noted otherwise.

### Accession numbers

In this work we used the genomic sequences of *P. falciparum*, accession numbers, in increasing order of chromosomes: NC_004325(1), NC_000910(2), NC_000521(3), NC_004318(1), NC_004326(1), NC_004327(2), NC_004328(1), NC_004329(1), NC_004330(1), NC_004314(1), NC_004315(1), NC_004316(2), NC_004331(1), NC_004317(1). Version numbers are given in brackets. For *Dictyostelium discoideum* we used M14628(1).

## Competing interests

The authors declare that they have no competing interests.

## Author contribution

MCSN wrote the Perl scripts, performed the calculations and prepared the figures. EFW and GW provided conceptual advice and supervised MCSN. All authors wrote the paper.
